# Supplementary material for: Diplopia Is Frequent and Associated with Motor and Non-Motor Severity in Parkinson’s Disease: Results from the COPPADIS Cohort at 2-Year Follow-Up
Source: Diagnostics (Basel). 2021 Dec 17;11(12):2380. doi: 10.3390/diagnostics11122380 (PMC8700703; doi:10.3390/diagnostics11122380)
Supplement: Supplementary file 1 [file diagnostics-11-02380-s001.zip › diagnostics-1477698-Supplementary.pdf]

Name (Last Name, First Name)	Location	Role	Contribution
Astrid Adarmes, Daniela	Hospital Universitario Virgen del Rocío, Sevilla, Spain	Site investigator	Evaluation of participants and/or data management
Almeria, Marta	Hospital Universitari Mutua de Terrassa, Terrassa, Barcelona, Spain	Site investigator	Neuropsychologist; evaluation of participants
Alonso Losada, Maria Gema	Hospital Álvaro Cunqueiro, Complejo Hospitalario Universitario de Vigo (CHUVI), Vigo, Spain	Site investigator/PI	Coordination at the center Evaluation of participants and/or data management
Alonso Cánovas, Araceli	Hospital Universitario Ramón y Cajal, Madrid, Spain	Site investigator	Evaluation of participants and/or data management
Alonso Frech, Fernando	Hospital Universitario Clínico San Carlos, Madrid, Spain	Site investigator	Evaluation of participants and/or data management
Alonso Redondo, Ruben	Hospital Universitario Lucus Augusti (HULA), Lugo, Spain	Site investigator/PI	Coordination at the center Evaluation of participants and/or data management
Aneiros Díaz, Ángel	Complejo Hospitalario Universitario de Ferrol (CHUF), Ferrol, A Coruña, Spain	Site investigator/PI	Coordination at the center Evaluation of participants and/or data management
Álvarez, Ignacio	Hospital Universitari Mutua de Terrassa, Terrassa, Barcelona, Spain	Site investigator	Evaluation of participants and/or data management
Álvarez Sauco, María	Hospital General Universitario de Elche, Elche, Spain	Site investigator/PI	Coordination at the center Evaluation of participants and/or data management
Arnáiz, Sandra	Complejo Asistencial Universitario de Burgos, Burgos, Spain	Site investigator	Evaluation of participants and/or data management
Arribas, Sonia	Hospital Universitari Mutua de Terrassa, Terrassa, Barcelona, Spain	Site investigator	Neuropsychologist; evaluation of participants
Ascunce Vidondo, Arancha	Complejo Hospitalario de Navarra, Pamplona, Spain	Site investigator	Evaluation of participants and/or data management
Aguilar, Miquel	Hospital Universitari Mutua de Terrassa, Terrassa, Barcelona, Spain	Site investigator	Evaluation of participants and/or data management
Ávila Rivera, Maria Asunción	Consorci Sanitari Integral, Hospital General de L´Hospitalet, L´Hospitalet de Llobregat, Barcelona, Spain	Site investigator/PI	Coordination at the center Evaluation of participants and/or data management
Bernardo Lambrich, Noemí	Hospital de Tortosa Verge de la Cinta (HTVC), Tortosa, Tarragona, Spain	Site investigator	Evaluation of participants and/or data management
Bejr-Kasem, Helena	Hospital de Sant Pau, Barcelona, Spain	Site investigator	Evaluation of participants and/or data management
Blázquez Estrada, Marta	Hospital Universitario Central de Asturias, Oviedo, Spain	Site investigator	Evaluation of participants and/or data management
Botí González, Maria Ángeles	Hospital Universitari Mutua de Terrassa, Terrassa, Barcelona, Spain	Site investigator	Neuropsychologist; evaluation of participants
Borrué, Carmen	Hospital Infanta Sofía, Madrid, Spain	Site investigator/PI	Coordination at the center Evaluation of participants and/or data management
Buongiorno, Maria Teresa	Hospital Universitari Mutua de Terrassa, Terrassa, Barcelona, Spain	Site investigator	Nurse study coordinator
Cabello González, Carolina	Complejo Hospitalario de Navarra, Pamplona, Spain	Site investigator	Scheduling of evaluations
Cabo López, Iria	Complejo Hospitalario Universitario de Pontevedra (CHOP), Pontevedra, Spain	Site investigator/PI	Coordination at the center Evaluation of participants and/or data management
Caballol, Nuria	Consorci Sanitari Integral,	Site investigator/PI	Coordination at the center

	Hospital Moisés Broggi, Sant Joan Despí, Barcelona, Spain.		Evaluation of participants and/or data management
Cámara Lorenzo, Ana	Hospital Clínic de Barcelona, Barcelona, Spain	Site investigator	Nurse study coordinator
Canfield Medina, Héctor	Complejo Hospitalario Universitario de Ferrol (CHUF), Ferrol, A Coruña, Spain	Site investigator	Evaluation of participants and/or data management
Carrillo, Fátima	Hospital Universitario Virgen del Rocío, Sevilla, Spain	Site investigator	Evaluation of participants and/or data management
Carrillo Padilla, Francisco José	Hospital Universitario de Canarias, San Cristóbal de la Laguna, Santa Cruz de Tenerife, Spain	Site investigator/PI	Coordination at the center Evaluation of participants and/or data management
Casas, Elena	Complejo Asistencial Universitario de Burgos, Burgos, Spain	Site investigator	Evaluation of participants and/or data management
Catalán, Maria José	Hospital Universitario Clínico San Carlos, Madrid, Spain	Site investigator/PI	Coordination at the center Evaluation of participants and/or data management
Clavero, Pedro	Complejo Hospitalario de Navarra, Pamplona, Spain	Site investigator	Evaluation of participants and/or data management
Cortina Fernández, A	Complejo Hospitalario Universitario de Ferrol (CHUF), Ferrol, A Coruña, Spain	Site investigator	Coordination of blood extractions
Cosgaya, Marina	Hospital Clínic de Barcelona, Barcelona, Spain	Site investigator	Evaluation of participants and/or data management
Cots Foraster, Anna	Institut d'Assistència Sanitària (IAS)—Instituí Cátala de la Salud. Girona, Spain	Site investigator	Evaluation of participants and/or data management
Crespo Cuevas, Ane	Hospital del Mar, Barcelona, Spain.	Site investigator	Evaluation of participants and/or data management
Cubo, Esther	Complejo Asistencial Universitario de Burgos, Burgos, Spain	Site investigator/PI	Coordination at the center Evaluation of participants and/or data management
De Deus Fonticoba, Teresa	Complejo Hospitalario Universitario de Ferrol (CHUF), Ferrol, A Coruña, Spain	Site investigator	Nurse study coordinator Evaluation of participants and/or data management
De Fábregues-Boixar, Oriol	Hospital Universitario Vall d´Hebron, Barcelona, Spain	Site investigator/PI	Coordination at the center Evaluation of participants and/or data management
Díez Fairen, M	Hospital Universitari Mutua de Terrassa, Terrassa, Barcelona, Spain	Site investigator	Evaluation of participants and/or data management
Dotor García-Soto, Julio	Hospital Universitario Virgen Macarena, Sevilla, Spain	Site investigator/PI	Evaluation of participants and/or data management
Erro, Elena	Complejo Hospitalario de Navarra, Pamplona, Spain	Site investigator	Evaluation of participants and/or data management
Escalante, Sonia	Hospital de Tortosa Verge de la Cinta (HTVC), Tortosa, Tarragona, Spain	Site investigator/PI	Coordination at the center Evaluation of participants and/or data management
Estelrich Peyret, Elena	Institut d'Assistència Sanitària (IAS)—Instituí Cátala de la Salud. Girona, Spain	Site investigator	Evaluation of participants and/or data management
Fernández Guillán, Noelia	Complejo Hospitalario Universitario de Ferrol (CHUF), Ferrol, A Coruña, Spain	Site investigator	Neuroimaging studies
Gámez, Pedro	Complejo Asistencial Universitario de Burgos, Burgos, Spain	Site investigator	Evaluation of participants and/or data management
Gallego, Mercedes	Hospital La Princesa, Madrid, Spain	Site investigator	Evaluation of participants and/or data management
García Caldentey, Juan	Centro Neurológico Oms 42, Palma de Mallorca, Spain	Site investigator/PI	Coordination at the center Evaluation of participants and/or data management
García Campos, Cristina	Hospital Universitario Virgen	Site investigator	Evaluation of participants

	Macarena, Sevilla, Spain		and/or data management
García Moreno, Jose Manuel	Hospital Universitario Virgen Macarena, Sevilla, Spain	Site investigator/PI (until MAR/21)	Coordination at the center Evaluation of participants and/or data management
Gastón, Itziar	Complejo Hospitalario de Navarra, Pamplona, Spain	Site investigator/PI	Coordination at the center Evaluation of participants and/or data management
Gómez Garre, María del Pilar	Hospital Universitario Virgen del Rocío, Sevilla, Spain	Site investigator	Genetic studies coordination
Gómez Mayordomo, Víctor	Hospital Clínico San Carlos, Madrid, Spain	Site investigator	Evaluation of participants and/or data management
González Aloy, Javier	Institut d'Assistència Sanitària (IAS)—Instituí Cátala de la Salud. Girona, Spain	Site investigator	Evaluation of participants and/or data management
González Aramburu, Isabel	Hospital Universitario Marqués de Valdecilla, Santander, Spain	Site investigator	Evaluation of participants and/or data management
González Ardura, Jessica	Hospital Universitario Lucus Augusti (HULA), Lugo, Spain	Site investigator/PI (until FEB/21)	Evaluation of participants and/or data management
González García, Beatriz	Hospital La Princesa, Madrid, Spain	Site investigator	Nurse study coordinator
González Palmás, Maria Josefa	Complejo Hospitalario Universitario de Pontevedra (CHOP), Pontevedra, Spain	Site investigator	Evaluation of participants and/or data management
González Toledo, Gabriel Ricardo	Hospital Universitario de Canarias, San Cristóbal de la Laguna, Santa Cruz de Tenerife, Spain	Site investigator	Evaluation of participants and/or data management
Golpe Díaz, Ana	Complejo Hospitalario Universitario de Ferrol (CHUF), Ferrol, A Coruña, Spain	Site investigator	Laboratory analysis coordination
Grau Solá, Mireia	Consorci Sanitari Integral, Hospital Moisés Broggi, Sant Joan Despí, Barcelona, Spain	Site investigator	Evaluation of participants and/or data management
Guardia, Gemma	Hospital Universitari Mutua de Terrassa, Terrassa, Barcelona, Spain	Site investigator	Evaluation of participants and/or data management
Hernández Vara, Jorge	Hospital Universitario Vall d´Hebron, Barcelona, Spain	Site investigator/PI	Coordination at the center Evaluation of participants and/or data management
Horta Barba, Andrea	Hospital de Sant Pau, Barcelona, Spain	Site investigator	Neuropsychologist; evaluation of participants
Idoate Calderón, Daniel	Complejo Hospitalario Universitario de Pontevedra (CHOP), Pontevedra, Spain	Site investigaor	neuropsychologist; evaluation of participants
Infante, Jon	Hospital Universitario Marqués de Valdecilla, Santander, Spain	Site investigator/PI	Coordination at the center Evaluation of participants and/or data management
Jesús, Silvia	Hospital Universitario Virgen del Rocío, Sevilla, Spain	Site investigator	Evaluation of participants and/or data management
Kulisevsky, Jaime	Hospital de Sant Pau, Barcelona, Spain	Site investigator/PI	Coordination at the center Evaluation of participants and/or data management
Kurtis, Mónica	Hospital Ruber Internacional, Madrid, Spain	Site investigator/PI	Coordination at the center Evaluation of participants and/or data management
Labandeira, Carmen	Hospital Álvaro Cunqueiro, Complejo Hospitalario Universitario de Vigo (CHUVI), Vigo, Spain	Site investigator	Evaluation of participants and/or data management
Labrador Espinosa, Miguel Ángel	Hospital Universitario Virgen del Rocío, Sevilla, Spain	Site investigator	Neuroimaging data analysis
Lacruz, Francisco	Complejo Hospitalario de Navarra, Pamplona, Spain	Site investigator	Evaluation of participants and/or data management
Lage Castro, Melva	Complejo Hospitalario Universitario de Pontevedra	Site investigator	Evaluation of participants and/or data management

	(CHOP), Pontevedra, Spain		
Lastres Gómez, Sonia	Complejo Hospitalario Universitario de Pontevedra (CHOP), Pontevedra, Spain	Site investigator	Neuropsychologist; evaluation of participants
Legarda, Inés	Hospital Universitario Son Espases, Palma de Mallorca, Spain	Site investigator/PI	Coordination at the center Evaluation of participants and/or data management
López Ariztegui, Nuria	Complejo Hospitalario de Toledo, Toledo, Spain	Site investigator/PI	Evaluation of participants and/or data management
López Díaz, Luis Manuel	Hospital Da Costa de Burela, Lugo, Spain	Site investigator	Evaluation of participants and/or data management
López Manzanares, Lydia	Hospital La Princesa, Madrid, Spain	Site investigator/PI	Coordination at the center Evaluation of participants and/or data management
López Seoane, Balbino	Complejo Hospitalario Universitario de Ferrol (CHUF), Ferrol, A Coruña, Spain	Site investigator	Neuroimaging studies
Lucas del Pozo, Sara	Hospital Universitario Vall d´Hebron, Barcelona, Spain	Site investigator	Evaluation of participants and/or data management
Macías, Yolanda	Fundación Hospital de Alcorcón, Madrid, Spain	Site investigator	Evaluation of participants and/or data management
Madrigal Lkhou, Elisabet	Complejo Asistencial Universitario de Burgos, Burgos, Spain	Site Investigator	Evaluation of participants and/or data management
Mata, Marina	Hospital Infanta Sofía, Madrid, Spain	Site investigator	Evaluation of participants and/or data management
Martí Andres, Gloria	Hospital Universitario Vall d´Hebron, Barcelona, Spain	Site investigator	Evaluation of participants and/or data management
Martí, Maria José	Hospital Clínic de Barcelona, Barcelona, Spain	Site investigator/PI	Coordination at the center Evaluation of participants and/or data management
Martínez Castrillo, Juan Carlos	Hospital Universitario Ramón y Cajal, Madrid, Spain	Site investigator /PI	Coordination at the center Evaluation of participants and/or data management
Martinez-Martin, Pablo	Centro Nacional de Epidemiología y CIBERNED, Instituto de Salud Carlos III. Madrid	Collaborator in statistical and methods analysis	Methods and statistical reviewer
McAfee, Darrian	University Maryland School of Medicine, Maryland United States of America	Collaborator in english style	English style reviewer
Meitín, Maria Teresa	Hospital Da Costa de Burela, Lugo, Spain	Site investigator	Evaluation of participants and/or data management
Menéndez González, Manuel	Hospital Universitario Central de Asturias, Oviedo, Spain	Site investigator/PI	Coordination at the center Evaluation of participants and/or data management
Méndez del Barrio, Carlota	Hospital Universitario Virgen del Rocío, Sevilla, Spain	Site investigator	Evaluation of participants and/or data management
Mir, Pablo	Hospital Universitario Virgen del Rocío, Sevilla, Spain	Site investigator/PI	Coordination at the center Evaluation of participants and/or data management
Miranda Santiago, Javier	Complejo Asistencial Universitario de Burgos, Burgos, Spain	Site investigator	Evaluation of participants and/or data management
Morales Casado, Maria Isabel	Complejo Hospitalario de Toledo, Toledo, Spain.	Site investigator	Evaluation of participants and/or data management
Moreno Diéguez, Antonio	Complejo Hospitalario Universitario de Ferrol (CHUF), Ferrol, A Coruña, Spain	Site investigator	Neuroimaging studies
Nogueira, Víctor	Hospital Da Costa de Burela, Lugo, Spain	Site investigator/PI	Coordination at the center Evaluation of participants and/or data management
Novo Amado, Alba	Complejo Hospitalario Universitario de Ferrol (CHUF), Ferrol, A Coruña, Spain	Site investigator	Neuroimaging studies

Novo Ponte, Sabela	Hospital Universitario Puerta de Hierro, Madrid, Spain.	Site investigator	Evaluation of participants and/or data management
Ordás, Carlos	Hospital Rey Juan Carlos, Madrid, Spain, Madrid, Spain.	Site Investigator	Evaluation of participants and/or data management
Pagonabarraga, Javier	Hospital de Sant Pau, Barcelona, Spain	Site investigator	Evaluation of participants and/or data management
Pareés, Isabel	Hospital Ruber Internacional, Madrid, Spain	Site investigator	Evaluation of participants and/or data management
Pascual-Sedano, Berta	Hospital de Sant Pau, Barcelona, Spain	Site Investigator	Evaluation of participants and/or data management
Pastor, Pau	Hospital Universitari Mutua de Terrassa, Terrassa, Barcelona, Spain	Site investigator	Evaluation of participants and/or data management
Pérez Fuertes, Aída	Complejo Hospitalario Universitario de Ferrol (CHUF), Ferrol, A Coruña, Spain	Site investigator	Blood analysis
Pérez Noguera, Rafael	Hospital Universitario Virgen Macarena, Sevilla, Spain	Site investigator	Evaluation of participants and/or data management
Planas-Ballvé, Ana	Consorci Sanitari Integral, Hospital Moisés Broggi, Sant Joan Despí, Barcelona, Spain	Site investigator	Evaluation of participants and/or data management
Planellas, Lluís	Hospital Clínic de Barcelona, Barcelona, Spain	Site investigator (until DEC/19)	Evaluation of participants and/or data management
Prats, Marian Ángeles	Institut d'Assistència Sanitària (IAS)—Instituí Cátala de la Salud. Girona, Spain	Site investigator	Evaluation of participants and/or data management
Prieto Jurczynska, Cristina	Hospital Rey Juan Carlos, Madrid, Spain, Madrid, Spain	Site investigator/PI	Coordination at the center Evaluation of participants and/or data management
Puente, Víctor	Hospital del Mar, Barcelona, Spain	Site investigator/PI	Coordination at the center Evaluation of participants and/or data management
Pueyo Morlans, Mercedes	Hospital Universitario de Canarias, San Cristóbal de la Laguna, Santa Cruz de Tenerife, Spain	Site investigator	Evaluation of participants and/or data management
Puig Daví, Arnau	Hospital de Sant Pau, Barcelona, Spain	Site einvestigator	Evaluation of participants and/or data management
Redondo, Nuria	Hospital La Princesa, Madrid, Spain	Site Investigator	Evaluation of participants and/or data management
Rodríguez Méndez, Luisa	Complejo Hospitalario Universitario de Ferrol (CHUF), Ferrol, A Coruña, Spain	Site investigator	Blood analysis
Rodríguez Pérez, Amparo Belén	Hospital General Universitario de Elche, Elche, Spain	Site investigator	Evaluation of participants and/or data management
Roldán, Florinda	Hospital Universitario Virgen del Rocío, Sevilla, Spain	Site investigator	Neuroimaging studies
Ruíz de Arcos, María	Hospital Universitario Virgen Macarena, Sevilla, Spain.	Site investigator	Evaluation of participants and/or data management
Ruíz Martínez, Javier	Hospital Universitario Donostia, San Sebastián, Spain	Site investigator	Evaluation of participants and/or data management
Sánchez Alonso, Pilar	Hospital Universitario Puerta de Hierro, Madrid, Spain	Site investigator	Evaluation of participants and/or data management
Sánchez-Carpintero, Macarena	Complejo Hospitalario Universitario de Ferrol (CHUF), Ferrol, A Coruña, Spain	Site investigator	Neuroimaging studies
Sánchez Díez, Gema	Hospital Universitario Ramón y Cajal, Madrid, Spain	Site investigator	Evaluation of participants and/or data management
Sánchez Rodríguez, Antonio	Hospital Universitario Marqués de Valdecilla, Santander, Spain	Site investigator	Evaluation of participants and/or data management
Santacruz, Pilar	Hospital Clínic de Barcelona, Barcelona, Spain	Site investigator	Evaluation of participants and/or data management
Santos García, Diego	CHUAC, Complejo Hospitalario Universitario de A Coruña	Coordinator of the Project	Coordination of the COPPADIS-2015

Segundo Rodríguez, José Clemente	Complejo Hospitalario de Toledo, Toledo, Spain	Site investigator	Evaluation of participants and/or data management
Seijo, Manuel	Complejo Hospitalario Universitario de Pontevedra (CHOP), Pontevedra, Spain	Site investigator/PI	Coordination at the center Evaluation of participants and/or data management
Sierra, María	Hospital Universitario Marqués de Valdecilla, Santander, Spain	Site investigator	Evaluation of participants and/or data management
Solano, Berta	Institut d'Assistència Sanitària (IAS)—Instituí Cátala de la Salud. Girona, Spain	Site investigator/PI	Coordination at the center Evaluation of participants and/or data management
Suárez Castro, Ester	Complejo Hospitalario Universitario de Ferrol (CHUF), Ferrol, A Coruña, Spain	Site investigator	Evaluation of participants and/or data management
Tartari, Juan Pablo	Hospital Universitari Mutua de Terrassa, Terrassa, Barcelona, Spain	Site investigator	Evaluation of participants and/or data management
Valero, Caridad	Hospital Arnau de Vilanova, Valencia, Spain	Site investigator	Evaluation of participants and/or data management
Vargas, Laura	Hospital Universitario Virgen del Rocío, Sevilla, Spain	Site investigator	Evaluation of participants and/or data management
Vela, Lydia	Fundación Hospital de Alcorcón, Madrid, Spain	Site investigator/PI	Coordination at the center Evaluation of participants and/or data management
Villanueva, Clara	Hospital Universitario Clínico San Carlos, Madrid, Spain	Site investigator	Evaluation of participants and/or data management
Vives, Bárbara	Hospital Universitario Son Espases, Palma de Mallorca, Spain	Site investigator	Evaluation of participants and/or data management
Villar, Maria Dolores	Hospital Universitario de Canarias, San Cristóbal de la Laguna, Santa Cruz de Tenerife, Spain	Site investigator	Evaluation of participants and/or data management
